# Somatosensory Amplification and Psychopathological Symptoms: The Mediating Roles of Meteoropathy and Meteosensitivity

**DOI:** 10.3390/brainsci16060575

**Published:** 2026-05-28

**Authors:** Krystian Konieczny, Monika Mak, Karol Karasiewicz, Krzysztof Wietrzyński, Karolina Rachubińska, Artur Reginia

**Affiliations:** 1 Department of Clinical Psychology and Psychoprophylaxis, Institute of Psychology, University of Szczecin, 71-017 Szczecin, Poland; 2 Department of Health Psychology, Pomeranian Medical University in Szczecin, 71-460 Szczecin, Poland; 3 Department of Social and Developmental Psychology, Institute of Psychology, University of Szczecin, 71-017 Szczecin, Poland; 4 Department of Psychiatry, Pomeranian Medical University in Szczecin, 71-460 Szczecin, Poland

**Keywords:** somatosensory amplification, meteoropathy, meteosensitivity, psychopathological symptoms, mediation model

## Abstract

**Background**: The mental health effects associated with weather conditions are diverse, indicating that individual susceptibility may play a significant role in this relationship. This study investigates whether somatosensory amplification is linked to meteosensitivity, meteoropathy, and psychopathological symptoms and whether meteoropathy and meteosensitivity serve as mediators in this relationship. **Methods**: This cross-sectional study included 523 participants from the general population. Psychopathological symptoms were assessed with the GFQ-58, somatosensory amplification with the SSAS-PL, and meteoropathy and meteosensitivity with the METEO-PL. Hierarchical linear regression was used to examine whether somatosensory amplification, meteoropathy, and meteosensitivity explained variance in psychopathological symptoms. A multiple mediator model was then estimated within the structural equation modeling framework using bootstrapping. The model was also tested separately for each of the 13 GFQ-58 symptom domains. **Results**: Somatosensory amplification accounted for a substantial portion of the variance in psychopathological symptoms (R^2^ = 0.247, *p* < 0.001). Upon incorporating meteoropathy and meteosensitivity, the explained variance increased to R^2^ = 0.571 (*p* < 0.001), with the increment being statistically significant (F(2, 521) = 32.12, *p* < 0.001). The indirect effect of somatosensory amplification via meteoropathy and meteosensitivity was significant (b = 0.851, 95% CI [0.661, 1.042], *p* < 0.001), and the direct effect also remained significant (b = 0.385, 95% CI [0.177, 0.594], *p* < 0.001), indicating partial statistical mediation. Significant indirect effects were also observed for poor social relationships, cognitive impairments, depressive symptoms, manic symptoms, anxiety symptoms, eating disorder symptoms, sleep problems, and somatic symptoms. **Conclusions**: Meteoropathy and meteosensitivity may partially account for the association between somatosensory amplification and psychopathological symptoms. The findings indicate that susceptibility to weather-related symptoms may be pertinent to the association between amplified bodily experiences and specific psychopathological symptoms.

## 1. Introduction

Associations between weather- and climate-related factors and mental health have been extensively studied. Overall, most evidence supports links between heat- and climate-related exposures and adverse mental health outcomes, although findings remain methodologically diverse and vary across exposure definitions, populations, and outcome measures [1,2]. An umbrella review identified that short-term increases in temperature were linked to mortality related to suicide or mental disorders, suicidal behavior, and hospital admissions due to suicidal behavior or mental disorders, although these associations were generally of small magnitude [1]. Similarly, a comprehensive analysis of psychiatric emergency department data from the United States revealed that days of extreme heat were correlated with increased visits across various diagnostic categories, including substance use disorders, anxiety- and stress-related disorders, somatoform disorders, mood disorders, and psychotic disorders. The same study also noted an increase in self-harm-related visits [3]. Consistent findings have also been reported in younger populations. In a large case-crossover study conducted in New York City, elevated daily minimum temperature was associated with increased mental health-related emergency department and hospital encounters among children, adolescents, and young adults [4]. Comparable findings have been reported in Europe. In Sweden, extremely high temperatures during the warm season were associated with increased psychiatric emergency visits, whereas in a Polish inpatient psychiatric setting, lower atmospheric pressure and foehn wind were linked to a higher number of aggressive incidents that necessitated coercive measures [5,6]. On a broader scale, a systematic review and meta-analysis indicated that heatwaves and extremely high temperatures are associated with an elevated risk of mental and behavioral disorders [7]. However, not all studies have aligned with these findings. A population-based study found that current meteorological parameters, including temperature, sunshine, and rainfall, were not associated with depressed mood and did not account for seasonal variations in major depressive disorder [8]. Furthermore, intensive longitudinal data obtained through mobile and wearable technologies suggest that seasonal and weather-related influences may exhibit distinct patterns across individuals and coincide with changes in physical activity [9]. Collectively, these findings imply that objective meteorological conditions alone do not fully explain the variability in mental health-related responses [8,9].

This heterogeneity underscores the variability among individuals in their susceptibility to weather changes and the degree to which they experience and report weather-induced physiological and psychological changes. In comprehensive clinical datasets, weather-related psychiatric conditions include somatoform disorders [3]. This finding emphasizes the need to consider weather-related reactivity not only in terms of exposure but also in the context of symptom manifestation. To describe these individual differences in weather-related reactivity, the literature uses the terms “meteosensitivity” and “meteoropathy” [10].

Meteosensitivity refers to biological susceptibility to the effects of atmospheric changes on the mind and body, whereas meteoropathy denotes the occurrence of specific symptoms or exacerbation of pre-existing conditions in response to such changes [10]. Meteoropathy is a syndrome comprising symptoms and pathological reactions that manifest when one or more meteorological factors, such as temperature, humidity, barometric pressure, or brightness, change gradually or suddenly within a given area [10,11]. Phenomenologically, it encompasses both somatic complaints and alterations in emotional functioning, including weakness, headache, joint and muscle pain, breathing difficulties, mood disturbances, irritability, palpitations, and pain in the sternum. These symptoms typically persist for one or two days, tend to diminish after the weather changes, and may recur with subsequent fluctuations [10]. Concurrently, empirical research on meteoropathy remains limited [12]. In women, both meteosensitivity and meteoropathy were positively correlated with cyclothymic and anxious temperaments and more weakly with depressive and irritable temperaments. Moreover, cyclothymic and anxious temperaments were related to meteoropathy both directly and indirectly through meteosensitivity [13]. In a person-centered study, personality heterogeneity differentiated meteosensitivity and meteoropathy, particularly among young adults, with higher levels of both constructs observed in individuals characterized by low emotional stability [14]. Meteoropathy was negatively correlated with emotional stability and positively correlated with the behavioral inhibition system (BIS), and BIS significantly mediated the association between emotional stability and meteoropathy [15]. These non-clinical findings are consistent with clinical observations. In patients with medication-overuse headache, meteoropathy intensity was positively correlated with depression and hopelessness [16]. In euthymic bipolar disorder, higher meteosensitivity and meteoropathy were associated with a history of suicide attempts, with affected individuals reporting more severe physical and psychological symptoms attributed to weather variations [17].

In the psychosomatic literature, somatosensory amplification is characterized as the propensity to perceive somatic sensations as intense, noxious, and disturbing [18]. According to Barsky’s conceptualization, this phenomenon involves bodily hypervigilance, a predisposition to focus on relatively weak and infrequent bodily sensations, and a tendency to interpret such sensations as abnormal or pathological rather than benign [18,19]. Empirical evidence suggests that heightened somatosensory amplification is correlated with increased symptom reporting, greater overall discomfort, and heightened social and vocational disability. It is also associated with depression, anxiety, and hostility, although it remains distinct from these dimensions of dysphoria [20]. Similarly, elevated scores on the Somatosensory Amplification Scale (SSAS) have been linked to depressive and anxiety disorders [19]. However, self-reported somatosensory amplification does not appear to solely reflect somatic sensitivity; rather, the SSAS seems to be more closely related to symptom reporting, general distress, and negative emotionality [21]. Recent theoretical perspectives have thus proposed that somatosensory amplification may be better understood not merely as bodily sensitivity but as an amplification of perceived threat to bodily integrity while remaining related, yet not identical, to health anxiety, hypochondriasis, and somatization [22].

Rather than reflecting objective meteorological conditions alone, meteosensitivity and meteoropathy concern subjectively experienced changes in well-being and symptom expression [10]. For this reason, both constructs appear particularly relevant to somatosensory amplification, understood as a propensity to perceive bodily sensations as intense, noxious, and disturbing [18] and to appraise them as threatening or pathological [22]. In this context, individuals with heightened somatosensory amplification may be more inclined to perceive and report weather-related bodily and emotional changes. Within the present model, this provides the rationale for conceptualizing meteosensitivity and meteoropathy as statistical mediators: meteosensitivity reflects perceived susceptibility to atmospheric changes, whereas meteoropathy reflects the symptomatic manifestation or exacerbation of complaints attributed to such changes. Together, these constructs describe the weather-related form in which amplified bodily experience may be associated with broader psychopathological symptom burden. Despite these converging observations, the relationship between these constructs has not been integrated into a unified model. Specifically, it remains to be elucidated whether meteoropathy and meteosensitivity may account for the association between somatosensory amplification and psychopathological symptoms. Accordingly, the present study investigated whether somatosensory amplification is associated with meteosensitivity, meteoropathy, and psychopathological symptoms in the general population and whether meteoropathy and meteosensitivity may serve as mediators of the relationship between somatosensory amplification and psychopathological symptoms.

## 2. Materials and Methods

### 2.1. Hypotheses

To answer the question of the role of somatosensory amplification, meteoropathy, and meteosensitivity in relation to psychopathological symptoms, the following hypotheses were formulated:

**H1.**  *Somatosensory amplification, meteoropathy, and meteosensitivity significantly explain psychopathological symptoms.*

**H2.**  *Meteoropathy and meteosensitivity explain a significant amount of variance in psychopathological symptoms in the context of somatosensory amplification.*

**H3.**  *Meteoropathy and meteosensitivity mediate the relationship between somatosensory amplification and psychopathological symptoms.*

### 2.2. Study Procedure

This study was conducted following the acquisition of approval no. KB 36/2024 from the Bioethics Committee of the Institute of Psychology, University of Szczecin (Poland).

Before data collection, an a priori sample size estimation was performed for the expected mediation model tested within the structural equation modeling framework. Monte Carlo simulations were conducted assuming a target statistical power of 1 − β = 0.80, a significance level of α = 0.05, and a small-to-moderate expected mediated effect. The required sample size was estimated to range from 97 to 149 participants.

For the study’s purposes, a comprehensive set of materials was prepared, including an informed consent form and a battery of research instruments. The study was conducted entirely using the paper-and-pencil method. Participants were recruited from the general population using snowball sampling and were eligible if they were 18 years of age or older, provided informed consent, and were able to complete the paper-and-pencil questionnaire battery. Individuals whose current cognitive or mental state prevented them from understanding the study information or completing the questionnaires were not included. The procedure was administered by psychologists. Prior to participation, each participant was informed about the study objectives, data processing procedures, data anonymization, and the right to withdraw from the study at any time without negative consequences.

### 2.3. Participants

The initial cohort of the study comprised N = 538 participants from the general population in Poland. Subsequent analyses excluded data from individuals identified as multivariate outliers, determined by the Mahalanobis distance from the centroid, using the criterion χ^2^(16) > 32, which corresponds to the 1% of the most extreme observations in a theoretical multivariate normal distribution. This procedure was applied to reduce the influence of atypical multivariate response profiles on regression and SEM estimates. Consequently, the final sample consisted of *n* = 523 participants. This sample included *n* = 342 (65%) women, *n* = 178 (34%) men, and *n* = 3 (0.6%) participants identifying as other. The age of the participants ranged from 18 to 76 years (M = 34.8, SD = 13.9, Md = 29, Q1 = 23, Q3 = 47). The detailed characteristics of the study sample are presented in Table 1.

### 2.4. Measures

**The General Functioning Questionnaire (GFQ-58) (Pol. Kwestionariusz Ogólnej Oceny Funkcjonowania (KOOF-58))** is a Polish instrument developed by Styła and Kowalski [23] and was used to assess general functioning and psychopathological symptoms across 13 domains informed by ICD-10 and DSM-IV-TR symptom categories. The instrument yields scores on the following scales: (1) poor functioning at work and home, (2) lack of entertainment, (3) poor social relationships, (4) cognitive impairments, (5) addictions, (6) positive psychotic symptoms, (7) depressive symptoms, (8) manic symptoms, (9) anxiety symptoms, (10) eating disorder symptoms, (11) sleep problems, (12) sexual problems, and (13) somatic symptoms. The GFQ-58 consists of 58 items referring to experiences during the previous 7 days and has demonstrated satisfactory reliability, with Cronbach’s α for the total score ranging from 0.89 to 0.92. The GFQ-58 was used because it provides a broad, multidomain assessment of psychopathological symptoms and functioning and has documented psychometric properties in Polish samples.

**The Somatosensory Amplification Scale (SSAS-PL)** is a self-report measure of somatosensory amplification, originally developed by Barsky et al. [20] and adapted for the Polish population by Konieczny et al. [24]. The Polish version comprises 9 items, and the participants responded using a 5-point Likert scale ranging from 1 (strongly disagree) to 5 (strongly agree). The SSAS-PL demonstrated satisfactory reliability, with Cronbach’s α ranging from 0.75 to 0.78 and McDonald’s ω of 0.78. The Polish validation study also supported a unidimensional structure and demonstrated convergent and discriminant validity. Measurement invariance was examined across validation subsamples comprising healthy adults, cardiology outpatients, and participants who were not hospitalized at the time of assessment but self-reported psychiatric diagnoses and/or current or past psychiatric treatment.

**The Meteoropathy Questionnaire (METEO-Q)** is a self-report instrument originally developed by Mazza et al. [10] to assess meteoropathy and meteosensitivity and adapted for the Polish population by Konieczny et al. [25]. The Polish version comprises 11 items and a checklist of 21 physical and psychological symptoms associated with meteorological changes. The participants responded using a 5-point Likert scale ranging from 0 (absent) to 4 (severe). Items 1–5 assess meteoropathy, and items 6–11 assess meteosensitivity. In the Polish validation study, the questionnaire supported a two-factor structure corresponding to meteoropathy and meteosensitivity; demonstrated high internal consistency, with Cronbach’s α = 0.94 for the total score and 0.86–0.93 for the subscales; and showed multigroup measurement invariance, as well as convergent and discriminant validity.

**Sociodemographic survey**—an original survey was conducted to gather data on the participants’ age, sex, health status, place of residence, and substance use.

### 2.5. Statistical Analysis

Statistical analyses were conducted on the final analytical sample of 523 participants. Descriptive statistics were used to characterize the study sample. Continuous variables were summarized using means, standard deviations, medians, and quartiles where appropriate, whereas categorical variables were summarized using frequencies and percentages.

To test H1 and H2, hierarchical linear regression analysis was conducted in two blocks. In the first block, psychopathological symptoms were predicted by somatosensory amplification. In the second block, meteoropathy and meteosensitivity were added to the model to determine whether they explained additional variance in psychopathological symptoms beyond somatosensory amplification. The change in explained variance between the two blocks was evaluated using the ΔR^2^ test. Multicollinearity among predictors in the regression models was assessed using the variance inflation factor (VIF), with values below 5 interpreted as indicating no evidence of substantial multicollinearity.

To test H3, a multiple mediation model was estimated within the structural equation modeling framework. Somatosensory amplification was specified as the predictor, meteoropathy and meteosensitivity as parallel mediators, and psychopathological symptoms as the outcome variable. Because meteoropathy and meteosensitivity are theoretically related METEO-Q dimensions, the covariance between their residual terms was freely estimated in the SEM mediation model. Indirect, direct, and total effects were estimated using 5000 bootstrap samples. Unstandardized coefficients, standardized coefficients, 95% confidence intervals, z values, and *p* values were reported.

To examine whether the mediation pattern differed across symptom domains, analogous mediation models were estimated separately for each of the 13 GFQ-58 symptom scales. Unless otherwise specified, the threshold for statistical significance was set at α = 0.05. Because these domain-specific analyses involved multiple testing, *p* values were adjusted using the Benjamini–Hochberg false discovery rate procedure, with the target FDR set at q = 0.05.

## 3. Results

To verify the first hypothesis, a hierarchical linear regression analysis was conducted in two blocks. In the first block, psychopathological symptoms were predicted by somatosensory amplification. In the second block, psychopathological symptoms were predicted by somatosensory amplification, meteoropathy, and meteosensitivity.

To test the second hypothesis, a ΔR^2^ test was conducted against the null hypothesis that the coefficients of determination for the two blocks (Blocks 1 and 2) did not differ. A *p* value below 0.05 was interpreted as indicating that meteoropathy and meteosensitivity explained a significant amount of additional variance in psychopathological symptoms in the context of somatosensory amplification. The results are presented in Table 2.

The results of the analysis suggest that somatosensory amplification explains approximately 24.7% of the variance in psychopathological symptoms (R^2^ = 0.247; *p* < 0.001). In Block 2, somatosensory amplification, meteoropathy, and meteosensitivity jointly explained approximately 57% of the variance in psychopathological symptoms (R^2^ = 0.571; *p* < 0.001). The difference was highly significant [F(2, 521) = 32.12; *p* < 0.001]. These results supported H1, indicating that somatosensory amplification was a significant predictor of psychopathological symptoms, and H2, indicating that meteoropathy and meteosensitivity explained approximately 34% of additional variance in psychopathological symptoms beyond somatosensory amplification. The regression weights are presented in Table 2. All VIF values were below the threshold of 5 adopted for these analyses, indicating no evidence of substantial multicollinearity among predictors. Additional robustness analyses by including sex, age, and education level as background covariates were conducted for the regression and SEM models and are reported in the Supplementary Materials (Tables S1 and S2). These analyses did not materially change the interpretation of the main findings.

**Figure 1:**
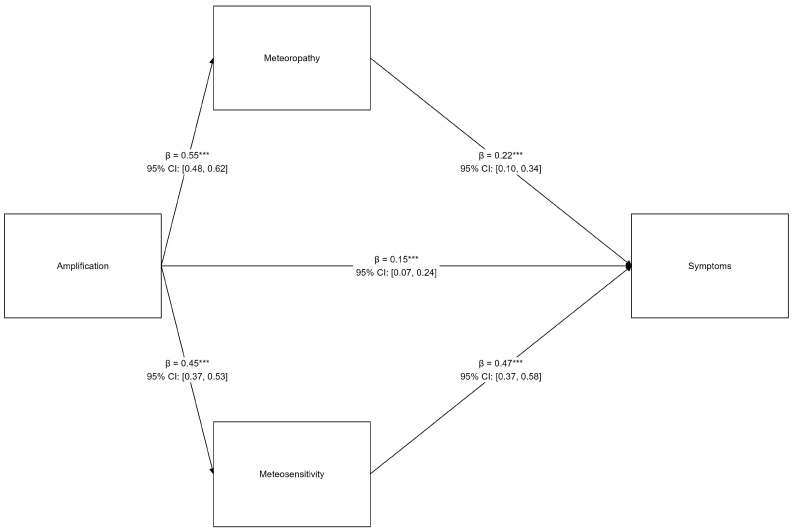
Mediation model for psychopathological symptoms. Note. *** *p* < 0.001.

In the next step, to verify H3, a mediation analysis based on a model similar to Model 7 proposed by Hayes and Preacher [26] was conducted within the SEM framework using 5000 bootstrap samples. Assumptions of normality of residuals and homoscedasticity were tested and met [27]. The results of the mediation analysis are presented in Table 3 and Figure 1.

The results were consistent with H3 and indicated a partial statistical mediation pattern, with a significant indirect association between somatosensory amplification and psychopathological symptoms through meteoropathy and meteosensitivity. The indirect effect of somatosensory amplification on symptoms through meteoropathy and meteosensitivity was statistically significant [b = 0.851; *p* < 0.001; 95% CI = (0.661, 1.042)] and explained approximately 68.8% of the total effect [b = 1.237; *p* < 0.001; 95% CI = (1.006, 1.468)]. The direct effect also remained statistically significant [b = 0.385; *p* < 0.001; 95% CI = (0.177, 0.594)].

To obtain more detailed results, we conducted a series of analogous mediation models for individual symptom domains, in contrast to the previous analysis that used an overall (summative) measure of psychopathology. Analyses were performed separately for each of the 13 scales. To account for multiple testing, *p* values were adjusted using the Benjamini–Hochberg false discovery rate procedure. The detailed results are presented in Table 4.

Results revealed significant indirect effects through meteoropathy and meteosensitivity for the following symptom domains after Benjamini–Hochberg correction: poor social relationships, cognitive impairments, depressive symptoms, manic symptoms, anxiety symptoms, eating disorder symptoms, sleep problems, and somatic symptoms.

## 4. Discussion

This study aimed to investigate the potential associations of somatosensory amplification, meteoropathy, and meteosensitivity with psychopathological symptoms, as well as to determine whether meteoropathy and meteosensitivity statistically mediate the relationship between somatosensory amplification and the severity of psychopathological symptoms. The findings were largely consistent with the study hypotheses, supporting the proposed pattern of associations and suggesting that susceptibility to weather-related symptoms may contribute to the relationship between amplified bodily experiences and psychopathological symptoms. Hypotheses H1 and H2 were substantiated by the data. Somatosensory amplification, meteoropathy, and meteosensitivity significantly explained variance in psychopathological symptoms. Furthermore, the inclusion of meteoropathy and meteosensitivity accounted for additional variance beyond that explained by somatosensory amplification. These results indicate that, in the regression model, meteoropathy and meteosensitivity accounted for additional variance in psychopathological symptom severity beyond somatosensory amplification alone. The supplementary robustness analyses further indicated that the main pattern of findings remained stable after accounting for demographic characteristics. Accordingly, somatosensory amplification is interpreted here as the body-focused symptom-appraisal component of the model, meteoropathy and meteosensitivity as weather-related susceptibility constructs, and psychopathological symptoms as the outcome domain. These findings align with H3, as the incorporation of meteoropathy and meteosensitivity significantly altered the relationship between somatosensory amplification and psychopathological symptoms. In the general model, the indirect pathway through meteoropathy and meteosensitivity was statistically significant, while the direct association between somatosensory amplification and psychopathological symptoms also remained significant. This pattern is consistent with partial statistical mediation. The specific indirect effects reported in Table 3 further indicated that the pathways through both meteoropathy and meteosensitivity were statistically significant. The estimate for the pathway through meteosensitivity was numerically larger than the estimate for the pathway through meteoropathy, although the formal comparison between these two indirect effects was not statistically significant. Although this mediation pattern should not be construed as evidence of a causal relationship, it suggests that meteoropathy and meteosensitivity may statistically account for part, but not all, of the association between amplified bodily experience and psychopathological symptoms. This interpretation is congruent with the original conceptualization of somatosensory amplification as the propensity to perceive ordinary bodily sensations as intense, disturbing, and noxious [19,20]. This aligns with subsequent research indicating that the construct should not be viewed merely as bodily hypersensitivity but rather in relation to symptom-focused appraisal, illness-related distress, and perceived threat to bodily integrity [18,21,22]. This interpretation is consistent with contemporary models of interoception, which position psychopathology not only at the level of bodily signal detection but also at the level of prediction, appraisal, and regulation of internal states [28,29,30].

The findings from the broader literature on weather, climate, and mental health provide crucial context for interpreting the present results. Studies conducted on the general population have demonstrated that the average meteorological effects on mood and depressive symptoms tend to be minor or inconsistent [8,31]. Concurrently, more recent comprehensive analyses suggest that elevated ambient temperatures, heatwaves, and other climatic exposures are linked to specific adverse mental and behavioral health outcomes, including suicide-related outcomes, increased psychiatric service utilization, and diminished mental health and well-being [1,2,7]. Such effect estimates may vary depending on definitions of exposure, sample characteristics, and outcome operationalization, including whether studies assess symptom severity, symptom exacerbation, service utilization, well-being, or diagnostic status. Reviews in this domain similarly highlight heterogeneous pathways and the probable influence of psychological and social moderators [32,33,34]. Overall, the present results align more closely with a vulnerability framework than with a model of direct and uniform weather effects on psychopathological symptoms.

In this context, the concepts of meteoropathy and meteosensitivity are particularly pertinent, as they encompass a level of functioning that aligns more closely with subjective experience than with mere objective meteorological exposure. The METEO-Q was specifically developed to evaluate these two interrelated yet distinct constructs, with meteosensitivity denoting biological susceptibility to atmospheric changes and meteoropathy referring to the occurrence or exacerbation of symptoms in response to such changes [10,17]. Previous research has associated meteoropathy and meteosensitivity with affective temperaments, personality heterogeneity, and clinical vulnerability [13,14,17]. Recent studies have further suggested that meteoropathy is inversely related to emotional stability and positively associated with BIS, with BIS partially mediating the relationship between emotional stability and meteoropathy [15]. Recent reviews have also highlighted that meteoropathy remains under-researched despite its potential clinical significance and extensive symptom profile, which includes anxiety, sleep-related complaints, pain-related symptoms, and depressive manifestations [12]. In this context, meteoropathy and meteosensitivity may be interpreted as statistical intermediate variables in the association between heightened bodily experiences and psychopathological symptom burden.

Empirical evidence indicates that the average effects of weather on individuals are generally modest, whereas variability in individual responses to weather can be considerable. Klimstra et al. [35] identified four distinct types of weather reactivity by correlating daily mood reports with objective weather data, suggesting that null or weak effects at the group level may obscure significant heterogeneity at the individual level. Similarly, Denissen et al. [31] reported minor average effects of weather on mood but noted substantial individual differences. These observations are pertinent to the current findings, as meteoropathy and meteosensitivity may exemplify this type of individual variability in weather-related experiences. Consequently, these findings align more closely with an individual differences model of weather reactivity rather than the assumption that atmospheric changes affect all individuals uniformly.

Somatosensory amplification should not be perceived as merely an increased sensitivity to bodily sensations. Research on interoception indicates that symptom reports are influenced not only by the precision of bodily signal detection but also by expectancy, attentional focus, response bias, stress reactivity, and the affective context. Petersen et al. [36] differentiated interoceptive accuracy from symptom-reporting bias. Wolters et al. [37] demonstrated that across somatic symptom disorder, illness anxiety disorder, and functional syndromes, the most consistent observation was not enhanced interoceptive accuracy but rather a pattern indicative of response bias and, in certain conditions, diminished accuracy. Eggart et al. [38] similarly concluded that major depressive disorder is associated with impaired interoceptive accuracy rather than a universally heightened body awareness. Schulz and Vögele [39] conceptualized interoception and stress as components of a bidirectional brain–body process pertinent to both physical and mental health symptoms. These findings suggest that bodily processing encompasses not only signal detection but also response bias, stress sensitivity, and affective contexts. Consequently, in the present context, somatosensory amplification may be understood not merely as heightened perception of bodily sensations but as a broader body-focused appraisal style that may facilitate the interpretation and reporting of weather-related bodily and affective changes. Meteoropathy and meteosensitivity may therefore mark a context-specific form in which this broader style of symptom processing is associated with psychopathological symptom burden. Symptom-specific mediation analyses further substantiate this interpretation by indicating selectivity rather than a broad, global mechanism. The domain-specific mediation models showed significant indirect associations through meteoropathy and meteosensitivity after Benjamini–Hochberg correction for the following GFQ-58 domains: poor social relationships, cognitive impairments, depressive symptoms, manic symptoms, anxiety symptoms, eating disorder symptoms, sleep problems, and somatic symptoms. This pattern aligns with the existing literature and can be linked to the somatosensory amplification component of the model. Interoceptive disturbances are relevant to this interpretation because somatosensory amplification involves intensified appraisal and processing of bodily sensations; such disturbances have consistently been associated with anxiety and depressive symptoms, while mental health burdens related to weather and heat have been linked to sleep disruption, fatigue, psychological stress, and decreased activity [2,28,29,40,41]. Notably, the literature does not support a uniform relationship between weather and all psychiatric symptom domains. Essers et al. [42] reported domain-specific rather than global associations with temperature exposure, and other clinical studies have specifically linked weather variables to depressive symptoms or selected psychiatric service outcomes rather than to a generalized symptom profile. Therefore, these findings are more consistent with domain-specific associations than with a single generalized association across all psychopathological symptom domains.

The findings presented herein may inform future clinically oriented research, although they are preliminary in nature. If these results are replicated, they suggest that individuals exhibiting high levels of somatosensory amplification may be particularly susceptible when amplified body-focused processing coincides with heightened subjective weather sensitivity. This interpretation aligns with prospective evidence from chronically ill individuals indicating that, under conditions of light-to-moderate ambient heat, symptom burden may be more pronounced when somatosensory amplification and negative expectations are elevated, whereas psychosocial resources may attenuate this burden [43]. Furthermore, this perspective is broadly consistent with intervention-oriented findings that demonstrate that interoception-based interventions can enhance interoceptive functioning, despite mixed outcomes regarding symptom reduction [44]. From this perspective, meteoropathy and meteosensitivity may serve as candidate indicators of a subgroup warranting further prospective investigation into interoceptive, self-regulatory, and symptom-focused interventions.

This study has several limitations. First, the cross-sectional design precludes causal inference, necessitating that the mediation pattern be interpreted as statistical rather than mechanistic. Second, all principal constructs were assessed via self-report, introducing the potential for shared method variance and limiting the ability to fully disentangle construct-specific associations from broader self-report tendencies, such as general distress-related symptom reporting. Third, the study did not incorporate objective meteorological exposure data, thereby preventing the differentiation of subjective weather sensitivity from actual environmental conditions. Fourth, the sample was recruited from the general population using snowball sampling and should therefore not be considered representative of the general population. This limits the generalizability of the findings. Nonetheless, these limitations suggest clear directions for future studies. Where feasible, prospective multimethod studies could combine validated questionnaires with one or more complementary data sources, such as ecological momentary assessment, objective weather indicators, and behavioral interoceptive measures, to examine whether the proposed pathway remains stable over time and whether it is particularly pertinent to depressive symptoms, anxiety symptoms, cognitive impairment, sleep problems, somatic symptoms, and eating disorder symptoms. Future research could also examine the physiological correlates of this pathway, building on evidence linking somatosensory amplification with long-latency neurophysiological processing and meteoropathy/meteosensitivity with autonomic, HPA-axis, vestibular, circadian, and sleep-related mechanisms. Future studies may also examine whether weather-related susceptibility and symptom expression are associated with broader constructs of environmental sensitivity, such as sensory processing sensitivity [45], which were not assessed in the present study. This approach would also facilitate a more direct examination of whether meteoropathy and meteosensitivity are best conceptualized as a transdiagnostic vulnerability process, a domain-specific sensitivity profile, or both.

## 5. Conclusions

Overall, the findings indicate that meteoropathy and meteosensitivity may partially account for the association between heightened bodily experiences and psychopathological symptoms. Given the cross-sectional design, this finding should be interpreted as a statistical mediation pattern rather than evidence of a causal or mechanistic pathway. Future multimethod studies should facilitate a more direct examination of whether meteoropathy and meteosensitivity are best conceptualized as a transdiagnostic vulnerability process, a domain-specific sensitivity profile, or both.

## Figures and Tables

**Table 1:** Sociodemographic characteristics of the study sample (N = 523).

Characteristic	N = 523
Sex	
Man	178 (34%)
Woman	342 (65%)
Other	3 (0.6%)
Age	18–76 (M = 34.8)
Education level	
Primary education (6–8 years)	12 (2.3%)
Lower secondary education (9 years)	4 (0.8%)
Vocational education (10–12 years)	21 (4.1%)
Secondary education (12–13 years)	197 (38%)
Post-secondary non-university education	32 (6.2%)
Higher education	252 (49%)
Place of residence	
Rural area	86 (17%)
Small town (<20,000 inhabitants)	79 (15%)
Medium-sized city (20,000–100,000 inhabitants)	124 (24%)
Large city (>100,000 inhabitants)	228 (44%)

Note. Frequencies do not add up to 523 because of missing responses for selected sociodemographic variables. All questionnaire scores used in the main regression and SEM analyses were complete. Educational categories refer to the highest completed level within the Polish educational system.

**Table 2:** Hierarchical linear regression models predicting psychopathological symptoms from somatosensory amplification, meteoropathy, and meteosensitivity.

Block	Predictor	b [95% CI]	β	z	*p*
1	Amplification	1.237 [1.011, 1.463]	0.50	10.74	<0.001
2	Amplification	0.388 [0.183, 0.593]	0.16	3.70	<0.001
	Meteoropathy	0.779 [0.355, 1.202]	0.22	3.60	<0.001
	Meteosensitivity	1.403 [1.075, 1.73]	0.48	8.38	<0.001

**Table 3:** Total, direct, and indirect effects in the mediation model linking somatosensory amplification with general psychopathological symptoms.

Label	b [95% CI]	β	z	*p*
mediated by Meteoropathy	0.31 [0.136, 0.483]	0.12	3.50	<0.001
mediated by Meteosensitivity	0.542 [0.371, 0.712]	0.22	6.23	<0.001
Difference	−0.232 [−0.519, 0.054]	−0.09	−1.59	0.112
Indirect	0.851 [0.661, 1.042]	0.34	8.76	<0.001
Direct	0.385 [0.177, 0.594]	0.15	3.62	<0.001
Total	1.237 [1.006, 1.468]	0.49	10.50	<0.001

**Table 4:** Total, direct, and indirect effects in mediation models for individual symptom domains.

Scale	Label	b [95% CI]	β	z	*p*	p*
Addictions						
	Direct	−0.027 [−0.059, 0.006]	−0.11	−1.60	0.109	0.139
	Indirect	0.011 [−0.008, 0.031]	0.05	1.16	0.244	0.311
	Total	−0.015 [−0.042, 0.011]	−0.06	−1.13	0.260	0.260
**Anxiety symptoms**						
	Direct	0.194 [0.096, 0.291]	0.24	3.91	<0.001	<0.001
	Indirect	0.131 [0.068, 0.193]	0.16	4.09	<0.001	<0.001
	Total	0.324 [0.245, 0.403]	0.40	8.02	<0.001	<0.001
**Cognitive impairments**						
	Direct	0.093 [0.047, 0.14]	0.23	3.95	<0.001	<0.001
	Indirect	0.072 [0.042, 0.102]	0.18	4.74	<0.001	<0.001
	Total	0.165 [0.127, 0.204]	0.41	8.33	<0.001	<0.001
**Sleep problems**						
	Direct	0.066 [−0.009, 0.141]	0.11	1.73	0.085	0.118
	Indirect	0.09 [0.043, 0.136]	0.15	3.76	<0.001	<0.001
	Total	0.155 [0.094, 0.217]	0.26	4.95	<0.001	<0.001
**Depressive symptoms**						
	Direct	0.134 [0.08, 0.188]	0.29	4.87	<0.001	<0.001
	Indirect	0.061 [0.028, 0.094]	0.13	3.59	<0.001	<0.001
	Total	0.195 [0.15, 0.24]	0.42	8.58	<0.001	<0.001
Poor functioning at work and at home						
	Direct	0.05 [−0.017, 0.118]	0.10	1.47	0.143	0.166
	Indirect	−0.002 [−0.041, 0.036]	−0.00	−0.12	0.908	0.908
	Total	0.048 [−0.007, 0.103]	0.09	1.71	0.087	0.102
Lack of entertainment						
	Direct	0.028 [−0.051, 0.107]	0.04	0.69	0.493	0.493
	Indirect	0.025 [−0.021, 0.07]	0.04	1.06	0.288	0.336
	Total	0.052 [−0.012, 0.117]	0.09	1.59	0.112	0.121
**Manic symptoms**						
	Direct	0.076 [0.002, 0.149]	0.13	2.03	0.043	0.075
	Indirect	0.059 [0.015, 0.103]	0.10	2.63	0.009	0.013
	Total	0.135 [0.074, 0.195]	0.23	4.38	<0.001	<0.001
**Eating disorder symptoms**						
	Direct	0.074 [0.039, 0.109]	0.25	4.16	<0.001	<0.001
	Indirect	0.04 [0.019, 0.062]	0.14	3.65	<0.001	<0.001
	Total	0.115 [0.086, 0.143]	0.39	7.83	<0.001	<0.001
Positive psychotic symptoms						
	Direct	0.045 [0, 0.091]	0.13	1.96	0.050	0.078
	Indirect	0.008 [−0.018, 0.035]	0.02	0.61	0.544	0.586
	Total	0.054 [0.017, 0.091]	0.15	2.85	0.004	0.006
**Poor social relationships**						
	Direct	0.068 [0.022, 0.115]	0.18	2.88	0.004	0.008
	Indirect	0.042 [0.013, 0.07]	0.11	2.87	0.004	0.007
	Total	0.11 [0.072, 0.148]	0.29	5.68	<0.001	<0.001
Sexual problems						
	Direct	0.064 [0.032, 0.097]	0.24	3.89	<0.001	<0.001
	Indirect	0.015 [−0.003, 0.034]	0.06	1.61	0.108	0.151
	Total	0.08 [0.053, 0.106]	0.30	5.87	<0.001	<0.001
**Somatic symptoms**						
	Direct	0.032 [−0.025, 0.088]	0.07	1.10	0.270	0.291
	Indirect	0.085 [0.048, 0.122]	0.19	4.49	<0.001	<0.001
	Total	0.116 [0.07, 0.162]	0.26	4.97	<0.001	<0.001

Note. p* = *p* value adjusted using the Benjamini–Hochberg false discovery rate procedure. Bolded scale names indicate symptom domains with significant indirect effects after FDR correction.

## Data Availability

The data presented in this study are available on request from the corresponding author due to legal reasons.

## Supplementary Materials

The following supporting information can be downloaded at: https://www.mdpi.com/article/10.3390/brainsci16060575/s1, Table S1: Regression coefficients for three-step models predicting psychopathological symptoms from demographic characteristics, somatosensory amplification, meteoropathy, and meteosensitivity; Table S2: Model summaries for three-step regression models predicting psychopathological symptoms from demographic characteristics, somatosensory amplification, meteoropathy, and meteosensitivity.
