# Supplementary material for: Somatosensory Amplification and Psychopathological Symptoms: The Mediating Roles of Meteoropathy and Meteosensitivity
Source: Brain Sci. 2026 May 28;16(6):575. doi: 10.3390/brainsci16060575 (PMC13297252; doi:10.3390/brainsci16060575)
Supplement: Supplementary file 1 [file brainsci-16-00575-s001.zip › brainsci-4293797-supplementary.pdf]

Supplementary Table S1. Regression coefficients for three-step models predicting psychopathological symptoms from demographic characteristics, somatosensory amplification, meteoropathy, and meteosensitivity

Aspect	Block	Predictor	b [95% CI]	β	z	p
Poor functioning at work and at home						
	1	Gender	-0.115 [-0.826, 0.596]	-0.02	-0.32	0.7518
		Age	0.054 [0.028, 0.08]	0.21	4.08	<0.001
	2	Gender	-0.366 [-1.092, 0.36]	-0.05	-0.99	0.32347
		Age	0.062 [0.035, 0.088]	0.24	4.59	<0.001
		Amplification	0.078 [0.022, 0.134]	0.15	2.72	0.00645
	3	Gender	-0.352 [-1.083, 0.38]	-0.05	-0.94	0.34595
		Age	0.063 [0.036, 0.089]	0.25	4.63	<0.001
		Amplification	0.075 [0.01, 0.141]	0.14	2.25	0.02426
		Meteoropathy	0.034 [-0.098, 0.167]	0.05	0.51	0.61055
		Meteosensitivity	-0.027 [-0.13, 0.076]	-0.04	-0.52	0.60196
Lack of entertainment						
	1	Gender	-0.26 [-1.097, 0.577]	-0.03	-0.61	0.54207
		Age	0.058 [0.027, 0.088]	0.20	3.71	<0.001
	2	Gender	-0.541 [-1.397, 0.315]	-0.07	-1.24	0.21532
		Age	0.066 [0.035, 0.097]	0.22	4.19	<0.001

Aspect	Block	Predictor	b [95% CI]	β	z	p
		Amplification	0.087 [0.021, 0.153]	0.14	2.58	0.00977
	3	Gender	-0.564 [-1.424, 0.295]	-0.07	-1.29	0.1981
		Age	0.068 [0.037, 0.1]	0.23	4.30	<0.001
		Amplification	0.059 [-0.018, 0.136]	0.10	1.50	0.13258
		Meteoropathy	0.102 [-0.054, 0.258]	0.12	1.29	0.19871
		Meteosensitivity	-0.027 [-0.148, 0.094]	-0.04	-0.44	0.66243
Poor social relationships						
	1	Gender	0.517 [0.001, 1.033]	0.10	1.96	0.04946
		Age	-0.024 [-0.043, -0.005]	-0.13	-2.50	0.01248
	2	Gender	0.191 [-0.323, 0.706]	0.04	0.73	0.46578
		Age	-0.014 [-0.033, 0.004]	-0.08	-1.53	0.12725
		Amplification	0.101 [0.061, 0.141]	0.27	4.99	<0.001
	3	Gender	0.094 [-0.416, 0.604]	0.02	0.36	0.71863
		Age	-0.016 [-0.034, 0.003]	-0.09	-1.64	0.10011
		Amplification	0.066 [0.021, 0.112]	0.18	2.84	0.00445
		Meteoropathy	0.021 [-0.071, 0.114]	0.04	0.45	0.65091
		Meteosensitivity	0.071 [0, 0.143]	0.16	1.95	0.05161
Cognitive impairments						

Aspect	Block	Predictor	b [95% CI]	β	z	p
	1	Gender	0.822 [0.288, 1.355]	0.15	3.02	0.00255
		Age	-0.055 [-0.074, -0.035]	-0.28	-5.52	<0.001
	2	Gender	0.36 [-0.155, 0.875]	0.07	1.37	0.17091
		Age	-0.041 [-0.06, -0.023]	-0.21	-4.35	<0.001
		Amplification	0.143 [0.104, 0.183]	0.35	7.06	<0.001
	3	Gender	0.267 [-0.231, 0.766]	0.05	1.05	0.29287
		Age	-0.038 [-0.056, -0.02]	-0.20	-4.15	<0.001
		Amplification	0.08 [0.035, 0.124]	0.20	3.50	<0.001
		Meteoropathy	0.171 [0.081, 0.262]	0.30	3.72	<0.001
		Meteosensitivity	-0.001 [-0.071, 0.069]	-0.00	-0.02	0.98112
Addictions						
	1	Gender	-0.438 [-0.783, -0.093]	-0.13	-2.49	0.0129
		Age	-0.007 [-0.02, 0.006]	-0.06	-1.10	0.27328
	2	Gender	-0.402 [-0.758, -0.046]	-0.12	-2.21	0.02692
		Age	-0.008 [-0.021, 0.005]	-0.07	-1.23	0.217
		Amplification	-0.011 [-0.039, 0.016]	-0.04	-0.79	0.42684
	3	Gender	-0.485 [-0.835, -0.136]	-0.15	-2.72	0.00649
		Age	-0.012 [-0.024, 0.001]	-0.10	-1.79	0.07305
		Amplification	-0.021 [-0.053, 0.01]	-0.08	-1.33	0.18339
		Meteoropathy	-0.078 [-0.141, -0.014]	-0.22	-2.40	0.01627

Aspect	Block	Predictor	b [95% CI]	β	z	p
		Meteosensitivity	0.104 [0.055, 0.153]	0.36	4.15	<0.001
Positive psychotic symptoms						
	1	Gender	0.112 [-0.375, 0.598]	0.02	0.45	0.65265
		Age	-0.026 [-0.044, -0.008]	-0.15	-2.86	0.00421
	2	Gender	-0.037 [-0.535, 0.461]	-0.01	-0.14	0.88494
		Age	-0.022 [-0.04, -0.004]	-0.13	-2.35	0.01877
		Amplification	0.046 [0.008, 0.085]	0.13	2.35	0.01889
	3	Gender	-0.157 [-0.642, 0.329]	-0.03	-0.63	0.52719
		Age	-0.028 [-0.045, -0.01]	-0.16	-3.09	0.00198
		Amplification	0.041 [-0.003, 0.084]	0.11	1.83	0.06747
		Meteoropathy	-0.156 [-0.244, -0.068]	-0.31	-3.48	<0.001
		Meteosensitivity	0.17 [0.101, 0.238]	0.41	4.87	<0.001
Depressive symptoms						
	1	Gender	1.118 [0.493, 1.744]	0.18	3.50	<0.001
		Age	-0.041 [-0.064, -0.018]	-0.18	-3.53	<0.001
	2	Gender	0.552 [-0.048, 1.151]	0.09	1.80	0.0712
		Age	-0.025 [-0.046, -0.003]	-0.11	-2.22	0.0265
		Amplification	0.176 [0.13, 0.222]	0.38	7.44	<0.001

Aspect	Block	Predictor	b [95% CI]	β	z	p
	3	Gender	0.464 [-0.128, 1.056]	0.08	1.53	0.1249
		Age	-0.023 [-0.044, -0.001]	-0.10	-2.07	0.0383
		Amplification	0.124 [0.071, 0.177]	0.27	4.57	<0.001
		Meteoropathy	0.122 [0.014, 0.229]	0.18	2.22	0.0262
		Meteosensitivity	0.018 [-0.065, 0.101]	0.03	0.42	0.6724
Manic symptoms						
	1	Gender	-0.786 [-1.577, 0.005]	-0.10	-1.95	0.0515
		Age	-0.062 [-0.091, -0.033]	-0.22	-4.18	<0.001
	2	Gender	-1.231 [-2.025, -0.436]	-0.16	-3.04	0.0024
		Age	-0.049 [-0.077, -0.02]	-0.17	-3.31	<0.001
		Amplification	0.138 [0.077, 0.199]	0.23	4.41	<0.001
	3	Gender	-1.355 [-2.144, -0.565]	-0.18	-3.36	<0.001
		Age	-0.048 [-0.077, -0.02]	-0.17	-3.31	<0.001
		Amplification	0.083 [0.013, 0.154]	0.14	2.31	0.021
		Meteoropathy	0.08 [-0.063, 0.223]	0.10	1.09	0.2749
		Meteosensitivity	0.067 [-0.044, 0.178]	0.10	1.18	0.2391
Anxiety symptoms						
	1	Gender	2.318 [1.256, 3.379]	0.22	4.28	<0.001

Aspect	Block	Predictor	b [95% CI]	β	z	p
		Age	-0.117 [-0.156, -0.078]	-0.30	-5.91	<0.001
	2	Gender	1.47 [0.435, 2.506]	0.14	2.78	0.00539
		Age	-0.092 [-0.13, -0.055]	-0.23	-4.82	<0.001
		Amplification	0.263 [0.183, 0.343]	0.32	6.45	<0.001
	3	Gender	1.152 [0.149, 2.155]	0.11	2.25	0.02444
		Age	-0.098 [-0.134, -0.061]	-0.25	-5.26	<0.001
		Amplification	0.167 [0.077, 0.257]	0.20	3.64	<0.001
		Meteoropathy	-0.016 [-0.198, 0.166]	-0.01	-0.17	0.86254
		Meteosensitivity	0.271 [0.13, 0.413]	0.28	3.76	<0.001
Eating disorder symptoms						
	1	Gender	0.603 [0.219, 0.988]	0.16	3.08	0.00209
		Age	-0.045 [-0.059, -0.031]	-0.32	-6.34	<0.001
	2	Gender	0.296 [-0.079, 0.671]	0.08	1.55	0.1219
		Age	-0.036 [-0.05, -0.023]	-0.26	-5.26	<0.001
		Amplification	0.095 [0.067, 0.124]	0.32	6.46	<0.001
	3	Gender	0.218 [-0.151, 0.588]	0.06	1.16	0.24618
		Age	-0.037 [-0.05, -0.023]	-0.26	-5.34	<0.001
		Amplification	0.062 [0.029, 0.095]	0.21	3.69	<0.001
		Meteoropathy	0.044 [-0.022, 0.111]	0.11	1.30	0.19325

Aspect	Block	Predictor	b [95% CI]	β	z	p
		Meteosensitivity	0.044 [-0.008, 0.096]	0.13	1.67	0.09584
Sleep problems						
	1	Gender	0.745 [-0.083, 1.572]	0.09	1.76	0.07782
		Age	-0.043 [-0.074, -0.013]	-0.15	-2.81	0.00502
	2	Gender	0.298 [-0.536, 1.131]	0.04	0.70	0.48397
		Age	-0.03 [-0.06, 0]	-0.10	-1.96	0.04964
		Amplification	0.139 [0.074, 0.203]	0.23	4.23	<0.001
	3	Gender	0.143 [-0.677, 0.963]	0.02	0.34	0.73258
		Age	-0.029 [-0.059, 0.001]	-0.10	-1.90	0.05771
		Amplification	0.061 [-0.012, 0.135]	0.10	1.63	0.10343
		Meteoropathy	0.146 [-0.003, 0.294]	0.17	1.92	0.05486
		Meteosensitivity	0.062 [-0.053, 0.178]	0.09	1.06	0.29018
Sexual problems						
	1	Gender	0.332 [-0.034, 0.697]	0.10	1.78	0.0754
		Age	-0.006 [-0.02, 0.007]	-0.05	-0.92	0.3559
	2	Gender	0.078 [-0.284, 0.44]	0.02	0.42	0.6731
		Age	0.001 [-0.012, 0.014]	0.01	0.17	0.8621
		Amplification	0.079 [0.051, 0.107]	0.30	5.52	<0.001
	3	Gender	0.071 [-0.292, 0.434]	0.02	0.38	0.7006

Aspect	Block	Predictor	b [95% CI]	β	z	p
		Age	0.003 [-0.011, 0.016]	0.02	0.39	0.6991
		Amplification	0.065 [0.032, 0.098]	0.25	3.91	<0.001
		Meteoropathy	0.058 [-0.008, 0.123]	0.15	1.71	0.0865
		Meteosensitivity	-0.02 [-0.071, 0.031]	-0.07	-0.78	0.4335
Somatic symptoms						
	1	Gender	1.007 [0.394, 1.62]	0.17	3.22	0.00127
		Age	-0.026 [-0.048, -0.003]	-0.12	-2.26	0.02382
	2	Gender	0.692 [0.074, 1.311]	0.12	2.19	0.02819
		Age	-0.017 [-0.039, 0.006]	-0.08	-1.45	0.14607
		Amplification	0.098 [0.05, 0.145]	0.22	4.01	<0.001
	3	Gender	0.486 [-0.108, 1.081]	0.08	1.60	0.10896
		Age	-0.02 [-0.042, 0.001]	-0.09	-1.85	0.06463
		Amplification	0.036 [-0.017, 0.089]	0.08	1.32	0.18573
		Meteoropathy	-0.012 [-0.119, 0.096]	-0.02	-0.21	0.83351
		Meteosensitivity	0.176 [0.092, 0.26]	0.33	4.12	<0.001
General score GFQ-58						
	1	Gender	5.975 [1.12, 10.83]	0.13	2.41	0.01586
		Age	-0.341 [-0.518, -0.163]	-0.20	-3.76	<0.001

Aspect	Block	Predictor	b [95% CI]	β	z	p
	2	Gender	1.361 [-3.255, 5.977]	0.03	0.58	0.56342
		Age	-0.205 [-0.372, -0.038]	-0.12	-2.41	0.01602
		Amplification	1.432 [1.076, 1.788]	0.40	7.88	<0.001
	3	Gender	-0.017 [-4.468, 4.433]	-0.00	-0.01	0.99387
		Age	-0.214 [-0.376, -0.053]	-0.12	-2.60	0.00937
		Amplification	0.899 [0.5, 1.298]	0.25	4.41	<0.001
		Meteoropathy	0.517 [-0.29, 1.323]	0.10	1.26	0.20918
		Meteosensitivity	0.908 [0.282, 1.535]	0.22	2.84	0.0045

Supplementary Table S2. Model summaries for three-step regression models predicting psychopathological symptoms from demographic characteristics, somatosensory amplification, meteoropathy, and meteosensitivity

Aspect	Block	R^2	ΔR^2	F(2, 356)	p	p*
Poor functioning at work and at home						
	1	0.05				
	2	0.07	0.02	5.75		
	3	0.07	0.00	0.24	0.788	0.788
Lack of entertainment						
	1	0.04				
	2	0.06	0.02	5.17		
	3	0.06	0.01	1.69	0.186	0.2
Poor social relationships						

Aspect	Block	R^2	ΔR^2	F(2, 356)	p	p*
	1	0.03				
	2	0.09	0.07	19.26		
	3	0.12	0.03	8.68	<0.001	<0.001
Cognitive impairments						
	1	0.10				
	2	0.21	0.11	38.64		
	3	0.27	0.06	22.54	<0.001	<0.001
Addictions						
	1	0.02				
	2	0.02	0.00	0.49		
	3	0.07	0.05	13.99	<0.001	<0.001
Positive psychotic symptoms						
	1	0.02				
	2	0.04	0.02	4.27		
	3	0.10	0.06	18.37	<0.001	<0.001
Depressive symptoms						
	1	0.06				
	2	0.19	0.13	42.93		
	3	0.22	0.03	10.68	<0.001	<0.001
Manic symptoms						

Aspect	Block	R^2	ΔR^2	$F(2, 356)$	p	p*
	1	0.06				
	2	0.11	0.05	15.08		
	3	0.13	0.02	7.32	<0.001	<0.001
Anxiety symptoms						
	1	0.13				
	2	0.22	0.09	32.19		
	3	0.28	0.06	21.81	<0.001	<0.001
Eating disorder symptoms						
	1	0.12				
	2	0.22	0.09	32.34		
	3	0.25	0.04	12.54	<0.001	<0.001
Sleep problems						
	1	0.03				
	2	0.08	0.05	13.84		
	3	0.12	0.04	12.77	<0.001	<0.001
Sexual problems						
	1	0.01				
	2	0.09	0.08	23.64		
	3	0.10	0.01	2.63	0.073	0.085
Somatic symptoms						

Aspect	Block	R^2	ΔR^2	F(2, 356)	p	p*
	1	0.04				
	2	0.08	0.04	12.45		
	3	0.16	0.08	25.90	<0.001	<0.001
General score GFQ-58						
	1	0.05				
	2	0.20	0.14	48.04		
	3	0.26	0.07	24.41	<0.001	<0.001

Note: p - p-values not corrected for multiple comparisons

p* - p-values with FDR Benjamini-Hochberg correction
